# Mpox on Instagram: Content Analytic Study

**DOI:** 10.2196/85379

**Published:** 2026-06-30

**Authors:** Elizabeth E Havron, Kylee Chenault, Danny Valdez, Rebecca F Houghton, Eric R Walsh-Buhi

**Affiliations:** 1 Department of Applied Health Science, Indiana University School of Public Health-Bloomington, 1025 E. 7th Street, Room 116, Bloomington, IN, 47405, United States, 1 812-855-4867

**Keywords:** digital health, social media, mpox, Instagram, content analysis

## Abstract

**Background:**

Mpox was declared a public health emergency of international concern in 2022. Instagram is widely used by age groups and communities disproportionately affected by mpox; yet platform-specific evidence on mpox information characteristics and engagement remains limited.

**Objective:**

The aim of this study is to characterize sources, content, and engagement features of mpox-related Instagram posts, to describe prevention and treatment framing, and to compare the top 10% most-liked posts with the remaining corpus.

**Methods:**

We retrieved English-language public Instagram posts via CrowdTangle containing “mpox” or “monkeypox” dated May 5, 2022, to January 17, 2023 (initial N=18,616). Using a pretested, deductive codebook adapted from prior Instagram health studies, 2 coders completed 2 pilot rounds; variables with low agreement were excluded. A randomized analytic sample of 1000 posts was coded for source type, content features, and prevention/treatment framing. Descriptive statistics were computed. For engagement contrasts, we compared the top 10% most-liked posts with the bottom 90% using tests of differences in independent proportions (mean differences [MD] with *P* values).

**Results:**

Most posts originated from organizations (760/1000, 76%) versus individuals (240/1000, 24%). Organizational sources most commonly included businesses (436/760, 57.4%) and news/media outlets (401/760, 52.8%); government (174/760, 22.9%), nonprofits (131/760, 17.3%), and health care organizations (70/760, 9.2%) were less frequent. About one-third of posts cited a source (344/1000, 34.4%), most often the World Health Organization (WHO) and Centers for Disease Control and Prevention (CDC)/other federal entity. Posts predominantly used illustrated images/infographics (827/1000, 82.7%); photos appeared in 47.3% (473/1000) and videos in 12.4% (124/1000) of posts. Prevention content appeared in 38.4% (384/1000) of posts, most commonly vaccination (684/1000, 68.5% of prevention posts), followed by avoiding close contact (145/1000, 14.5%), avoiding contact with objects (83/1000, 8.3%), abstaining from sexual activity (76/1000, 7.6%), and condom use (13/1000, 1.3%); 28.9% (289/1000) of prevention posts noted barriers. Treatment mentions were uncommon (25/1000, 2.5% traditional biomedical; 2/1000, 0.2% alternative). Compared with the bottom 90%, the top 10% most-liked posts (1) were more likely to originate from public figures/celebrities among individuals (MD=−0.591; *P*<.001) and from businesses (MD=−0.299; *P*<.001) or news/media (MD=−0.350; *P*<.001) among organizations; (2) were less likely to be from government (*P*<.001) , nonprofit (*P*=.006), or health care organizations (*P*=.005); and (3) more often included nonmoving images (MD=−0.119; *P*=.024), visible lesion depictions (MD=−0.081; *P*=.035), prevalence mentions (MD=−0.180; *P*<.001), and citations (MD=−0.162; *P*=.001).

**Conclusions:**

During the initial outbreak period, the highly engaged mpox content on Instagram skewed toward posts by public figures and news/business accounts and toward static, citation-bearing visuals that included prevalence context and occasionally lesion imagery. Public-health communicators seeking reach on Instagram should prioritize clear static infographics with explicit source citation and epidemiologic context and consider copublishing with trusted creators and news outlets, while addressing access barriers highlighted in prevention posts.

## Introduction

Mpox, a rare zoonotic disease caused by the “Monkeypox” virus, a species of the genus *Orthopoxvirus*, was declared a public health emergency of international concern on July 23, 2022 by the World Health Organization (WHO) [1,2]. Since the May 2022 outbreak, the disease has spread to 114 nonendemic countries. As of January 2024, the latest mpox outbreak has resulted in 99,518 cases globally and 32,063 cases in the United States [3]. A great majority of confirmed cases, about 82%, are people who identify as bisexual or who are men who have sex with men [4]. These data depict a disproportionate infection rate among men who have sex with men, which has accompanied concerns about even greater numbers of infections within this community and other priority populations.

Additionally, for many adults, social media represents a primary outlet for engaging with current events, including those pertaining to health concerns such as mpox [5]. Social media is used by almost 60% of the world and reaches a diverse population [6]. Instagram, in particular, offers users exposure to a variety of media, including photos, videos, and infographics, and has 3 billion monthly active users and over 500 million daily active users [7]. In 2025, 80% of Americans aged 18 to 29 years and 62% of Americans aged 30 to 49 years reported Instagram use as a social media platform [8]. In terms of race and ethnicity, 45% of White individuals, 52% of Black individuals, and 59% of Hispanic individuals report using Instagram [8]. Social media is used more by people who identify as lesbian, gay, bisexual, transgender, and queer/questioning (LGBTQ+) than by people who identify as heterosexual [9]. These data correlate with the demographics of reported mpox cases in the United States. According to the Centers for Disease Control and Prevention (CDC), the median age of people diagnosed with mpox was 35 years, and 94% of cases have been among men who have sex with men; 41%, 26%, and 28% of people diagnosed with mpox are White, Black, and Hispanic, respectively [10]. Furthermore, there is insufficient evidence on the use of Instagram as a modality for spreading information and awareness specifically regarding mpox. As such, a deep review of mpox-related Instagram content, as an example of a modern public health crisis unfolding online [11], is both timely and warranted, especially given that mpox knowledge and awareness in the United States are very low [12], and the populations with higher mpox infection prevalence are also among those with higher proportions utilizing Instagram.

The purpose of this study was to explore mpox-related content conveyed on Instagram and intentionally focus on the initial outbreak period (May 2022 to January 2023) to capture early-stage infodemic dynamics, which are critical for understanding how information ecosystems emerge during public health crises. Our study was guided by the following research questions:

- RQ1: What are the source and content characteristics of Instagram posts related to mpox?
- RQ2: What mpox causes and solution framing are conveyed in Instagram posts (ie, what is the content on Instagram related to mpox causes, treatment, and prevention)?
- RQ3: What are the content characteristics of the top 10% most-liked posts on mpox within the sample?

Findings from this work stand to inform the general digital ecosystem pertaining to mpox on Instagram. Furthermore, the contextualization of these findings may similarly suggest the possibility for future digital health interventions choosing to leverage social media outlets to distribute accurate, scientifically vetted information.

## Methods

### Data

Data for this study were collected from Instagram, a Metaverse photo-sharing social media platform. To source Instagram posts, our team utilized CrowdTangle Search, a public insights and third-party web-scraping tool with access to Metaverse data. We leveraged CrowdTangle to filter through all publicly accessible English-language posts that contained one of 2 queries (similar to a library search): (1) “mpox,” the updated CDC-supported name for the virus, or (2) “monkeypox,” the former name of the virus. Data were collected for posts dated from May 5, 2022, through January 17, 2023. English-language posts were identified based on caption language. This study used publicly available social media data. No private or identifiable user data were collected or reported. All data were analyzed in aggregate, further described below, and no individual accounts are identifiable in this study. A total of 18,616 posts met these inclusion criteria and were downloaded for use in 2 iterative pilot coding rounds, and for the final randomized data sample, as specified below. As of August 14, 2024, CrowdTangle has been shut down and replaced by the Meta Content Library, which has more limited access.

### Analyses

Our study employed a manual qualitative deductive coding procedure to review the data. “Deductive coding” refers to a typology of qualitative research where a researcher begins with a theoretical framework, sets of hypotheses, or previously established coding schemas. For our study, we relied on a pretested codebook that had been applied to previous Instagram-related research studies, including pre-exposure prophylaxis for HIV prevention and skin cancer [13,14]. We adapted this codebook to reflect discourse pertaining to mpox, which was further stratified along several key dimensions, including source characteristics; mpox risk factors; prevention methods; treatment methods, benefits, and barriers; stigma; and citation attributions. These dimensions reflected our coding schema. Each post included for analysis was compared against our rubric. Prior to analyzing the final dataset, Cohen κ statistics were analyzed for interrater reliability among our human coders using RStudio (Posit, PBC, formerly known as RStudio, Inc). The full codebook with associated Cohen κ values can be found in Multimedia Appendix 1. For the final analysis, IBM SPSS 26 was used to analyze descriptive statistics. For RQ3, specifically, tests for statistical differences (ie, Wald H0 differences in proportion) between independent sample proportions were run between the top 10% most-liked posts in the sample and those in the bottom 90% of liked posts. The “top 10% most-liked posts” were defined as the subset of posts in our analytic sample (N=1000) with the highest number of likes. Specifically, posts were ranked by total like count, and the top decile (n=100 posts) was classified as “most-liked,” while the remaining 90% (n=900 posts) served as the comparison group. This approach allowed us to examine differences in content characteristics between highly visible posts and the broader corpus. Wald tests were used due to a large sample size and suitability for comparing independent proportions. Sparse categories were interpreted cautiously. Mean differences (MDs) and associated *P* values are reported.

### Data Coding

Two human coders examined profile source and message content characteristics. The 2 coders and a moderator conducted 2 iterative pilot rounds using the most chronologically recent posts in the downloaded CrowdTangle dataset, where both coders coded the same set of posts and were assessed for interrater reliability. After the second round, among 72 variables and 63 out of 70 coded posts that met inclusion criteria, Cohen κ statistics ranged from −0.17 to 1.0, with a mean κ of 0.70 and median κ of 0.83, indicating substantial to perfect agreement across the majority of variables. Variables with none to slight agreement after the second round of pilot coding included posts’ cues to action, portrayal of mpox risk factors, diagnosis- and treatment-related content, and the extent to which posts conveyed the seriousness of mpox. Variables with low interrater reliability (eg, cues to action, risk factors, and seriousness) were excluded to preserve analytic validity. Prevention-related variables were retained and serve as proxies for risk-related content.

### Sample

Following the 2 pilot rounds, a total of 1200 Instagram posts were randomized and split between the 2 independent human coders with the intention of achieving a final dataset of 1000 for analyses. A sample of 1000 posts was selected to ensure adequate representation across content features, and this sample size is consistent with prior Instagram-based content analyses using manual (human) coding approaches [13,14]. Coder 1 coded 499 posts and Coder 2 coded 501 posts that ultimately met the inclusion criteria. We excluded posts that did not mention mpox or were not written in English. Coded variables included, but were not limited to, source characteristics (ie, organization type [eg, business and nonprofit] and individual type [eg, physician and journalist]) as well as mention of mpox causes, prevention methods, and treatment. The determination of the type of individual or organization behind the profile was based on self-described information in the account biography and/or post content (eg, credentials provided such as MD, journalist, or organizational affiliation), consistent with prior Instagram content analyses [13,14]. Photographic content was coded based on visible features within the image (eg, mpox lesion depiction, presence of individuals, and contextual clues), following predefined codebook criteria. Hashtags were extracted from post captions and analyzed descriptively. Frequencies of hashtag usage were computed, and the top 10 most common hashtags were identified. The full codebook can be found in Multimedia Appendix 1.

### Ethical Considerations

This study utilized publicly available Instagram data and did not involve direct interaction or intervention with human participants. The study was reviewed by the Institutional Review Board of Indiana University-Bloomington, affiliated with the last author and was determined not to constitute human subjects research.

## Results

### RQ1: What Are the Source and Content Characteristics of Instagram Posts Related to Mpox?

#### Sources

Roughly three-quarters (n=760, 76%) of the posts in this sample originated from organizational Instagram accounts, while the remaining quarter (n=240, 24%) originated from the accounts of individuals. Among organizations, a majority were classified as businesses (436/760, 57.4%) and/or news outlets (401/760, 52.8%). Posts originating from state, federal, or foreign equivalent comprised 22.9% (174/760) of organizational posts, followed by government entities (174/760, 22.9%), nonprofit organizations (131/760, 17.3%), health care organizations (70/760, 9.2%), and school districts (19/760, 2.5%). Among posts by individual accounts, almost half were made by public figures (107/240, 44.6%), followed by those who identified as parents (37/240, 15.4%), journalists (15/240, 6.3%), and physicians (13/240, 5.4%). Health workers, health educators, teachers, children, and epidemiologists each represented less than 3% (7/240) of the sample, as specified in Table 1.

**Table 1.** Source characteristics of Instagram posts regarding mpox^a^.

Source characteristics	All, n (%)	Most liked, n (%)	Less liked, n (%)	Mean difference (SE)	*P* value
Of posts made by an individual (N=240 for all; n=29 for top 10% most liked; n=211 for less liked), the bio/profile or post content mentioned...					
Public figure/celebrity with a minimum of 20,000 followers or having a verified account	107 (44.6)	28 (96.6)	79 (37.4)	−0.591 (0.048)	<.001
Being a parent (eg, mother or father)	37 (15.4)	1 (3.4)	36 (17.1)	0.136 (0.043)	.06
Being a journalist or member of the press	15 (6.3)	1 (3.4)	14 (6.6)	0.032 (0.007)	.60
Being a physician (eg, medical doctor, MD^b^, DO^c^, resident)	13 (5.4)	1 (3.4)	12 (5.7)	0.022 (0.037)	.62
Being a health worker	6 (2.5)	1 (3.4)	5 (2.4)	−0.011(0.035)	.73
Being a health educator	3 (1.3)	1 (0)	3 (1.4)	0.014 (0.008)	.52
Being a teacher, educator, or other school official	3 (1.3)	1 (3.4)	2 (0.9)	−0.025 (0.035)	.26
Being a child (eg, son or daughter)	2 (0.8)	0 (0)	2 (0.9)	0.009 (0.007)	.60
Being an epidemiologist	2 (0.8)	0 (0)	2 (0.9)	0.009 (0.007)	.60
Of post made by an organization (N=760 for all posts; n=71 for top 10% most liked; n=689 for less liked), the bio/profile/affiliated website mentioned...					
Business (eg, company, franchise, store, product, or service)	436 (57.4)	60 (84.5)	376 (54.6)	−0.299 (0.047)	<.001
News or media organization	401 (52.8)	60 (84.5)	341 (49.5)	−0.350 (0.047)	<.001
City, state, or federal government	174 (22.9)	2 (2.8)	172 (25)	0.222 (0.026)	<.001
Nonprofit/advocacy group	131 (17.3)	4 (5.6)	127 (18.5)	0.129 (0.031)	.006
Health care organization	70 (9.2)	0 (0)	70 (10.2)	0.102 (0.012)	.005
School or school district	19 (2.5)	2 (2.8)	17 (2.5)	−0.003 (0.021)	.86

a (1) Source characteristics do not add up to 100% due to information being unavailable on the individual poster’s Instagram biography. (2) Source characteristics exceed 100% because an organization may be included under multiple categories (eg, a business and a health care organization).

b MD: Doctor of Medicine.

c DO: Doctor of Osteopathic Medicine.

#### Cited Content

Among the full sample, about one-third (344/1000, 34.4%) of posts directly cited a source for their content. Of those posts, citations of the WHO were most prevalent, with over one-third of posts with citations referencing the WHO (120/344, 34.9%). The CDC, other federal entities, or foreign equivalents were the next most referenced (94/344, 27.3%). Almost one-quarter (80/344, 23.3%) of the sampled posts cited a medical doctor, followed by government officials (77/344, 22.5%), state or local health departments (60/344, 17.4%), and members of the research community (Table 2). Only a handful of posts cited a personal account from an individual who mentioned either firsthand or secondhand experience with mpox.

**Table 2.** Source attribution in mpox-related Instagram post citations.

Source attribution: The post cites information from...	All (N=344), n (%)	Most liked (n=49), n (%)	Less liked (n=295), n (%)	Mean difference (SE)	*P* value
Centers for Disease Control or other federal level or foreign equivalent	94 (27.3)	17 (38.8)	77 (26.1)	−0.086 (0.073)	.21
A medical doctor (physician)	80 (23.3)	11 (22.4)	69 (23.4)	0.009 (0.065)	.89
Government officials (eg, senators, governors, and representatives) or from political organizations	77 (22.5)	12 (22.4)	65 (22.1)	−0.024 (0.066)	.71
A state or local health department	60 (17.5)	5 (10.2)	55 (18.6)	0.084 (0.049)	.15
World Health Organization	120 (34.9)	19 (38.8)	101 (34.2)	−0.045 (0.075)	.54
A member of the research community (eg, researchers and scientists)	28 (8.2)	4 (8.2)	24 (8.1)	0.000 (0.042)	.99
Personal account of an individual who mentioned a firsthand experience with monkeypox (N=1000, full sample)	3 (0.3)	0 (0)	3 (0.3)	0.003 (0.002)	.57
Personal account of an individual who mentioned a secondhand experience with monkeypox (N=1000, full sample)	2 (0.2)	0 (0)	2 (0.2)	0.002 (0.002)	.64

#### Content Type

The majority of all posts (827/1000, 82.7%) related to mpox provided an image with an illustration (eg, infographic or text on the image, including data visualizations). Almost half (473/1000, 47.3%) displayed a photograph. Videos were depicted in 12.4% (124/1000) of posts overall (Table 3).

**Table 3.** Definitions, examples, and frequencies of textual content characteristics/variables regarding mpox-related messages.

Message characteristic	Definition	Example post description or quoted text	All posts (N=1000), n (%)	Top 10% most liked (n=100), n (%)	Bottom 90% less liked (n=900), n (%)
Profile type: individual	Profile represents a single person, not an organization or cause.	Profile of Senator Bill Frist, according to the profile biography	240 (24)	29 (29)	211 (23.4)
Profile type: organization	Profile represents an organization, not a single person.	Official profile of the San Bernardino County Public Health	760 (76)	71 (71)	689 (76.6)
Specified location	Location is specified anywhere in the post, either geotagged or referenced in text.	San Bernardino County Public Health logo appears within image	622 (62.3)	45 (45)	577 (57.7)
Content type: photo	Post includes some sort of photo (nonmoving photo or image and snapshot).	—^a^	473 (47.3)	58 (58)	415 (46.1)
Content type: video	Post includes some sort of video (eg, boomerangs, GIFs^b^, and IGTV^c^).	—	124 (12.4)	10 (10)	114 (12.7)
Content type: image with text	Post includes an illustration, text on image, and/or charts, etc (not specifically photo or video).	—	827 (82.7)	87 (87)	740 (82.2)
Race hashtag included	Post includes race-associated tags (eg, #Black and #Latino) or any mention of race in the text of the post.	“#BlackQueer #BlackQueerJoy”	32 (3.2)	1 (1)	31 (3.4)
Gender hashtag included	Post includes gender-associated tags (eg, “man,” “boy,” “woman,” “girl,” “trans,” and “transgirl"). If yes, copy and paste text.	“#empoweringmen #empoweringwomen”	15 (1.5)	1 (1)	14 (1.6)
Sexuality hashtag included	Post includes sexuality tags (eg, ”gay,” “homosexual,” “msm” [men who have sex with men], and “LGBTQ+”^d^). If yes, copy and paste text.	“#LGBTQcommunity #QueerCouple #BlackQueerJoy #BlackQueer #GayCouple #GayLove #GayPride #QueerpPride#LGBTQPride #LGBTQLove”	42 (4.2)	4 (4)	38 (4.2)
Mpox visual depictions	Image displays mpox on a specific body part (ie, face, chest, arms, hands, legs, and genitalia).	—	157 (15.7)	22 (22)	134 (14.9)
Monkey	The image or video displays a picture of a monkey or monkeys.	—	41 (4.1)	6 (6)	35 (3.9)
Mpox prevention methods	The post mentions mpox prevention methods?	“Avoid close, skin-to-skin contact with infected people. Don’t share cups, utensils, bedding, towels, clothing, upholstered seating, or other textiles with an infected person. Wash your hands or use an alcohol-based hand sanitizer. Get the smallpox vaccine if you are at high risk for infection”	384 (38.4)	36 (36)	348 (38.7)
Prevention: condoms (n=384)	Post promotes the use of condoms as a form of mpox prevention.	“Because of current uncertainties about transmission, wear a condom for 12 weeks even after you have fully recovered”	5 (1.3)	1 (2.7)	4 (1.1)
Prevention: vaccine promotion (n=384)	Post promotes getting the vaccine as a form of mpox prevention.	“Get the smallpox vaccine if you are at high risk for infection”	263 (68.5)	21 (56.8)	242 (69.5)
Prevention: avoiding close contact (n=384)	Post promotes avoiding close contact with infected persons as a form of mpox prevention.	“Avoid close, skin-to-skin contact with infected people”	56 (14.6)	6 (16.2)	50 (14.4)
Prevention: abstinence (n=384)	Post promotes abstaining from kissing, hugging, cuddling, and any other sexual activity as a form of mpox prevention.	“Stop or limit gay orgy attendance”	29 (7.6)	3 (8.1)	26 (7.5)
Prevention: objects (n=384)	Avoiding contact with objects (including bedding, sex toys, towels, and clothing) or sharing food/drink/other that an infected person has touched/used.	“Don’t share cups, utensils, bedding, towels, clothing, upholstered seating, or other textiles with an infected person”	32 (8.3)	4 (10.8)	28 (8)
Benefits of prevention (n=384)	The post mentions the benefits of a prevention method (eg, condoms also protect against sexually transmitted infections, pregnancy, etc).	“Intradermal administration of the JYNNEOS vaccine allows providers to administer up to five separate doses from a single vial. This method will help us protect more people from getting #monkeypox with our existing supply of vaccines”	28 (7.3)	1 (2.7)	27 (7.1)
Barriers to prevention (n=384)	The post mentions barriers to the prevention of mpox (ie, lack of knowledge on prevention methods, vaccines are unavailable).	“ACAM2000 has a larger stockpile but more restrictions for administration, whereas JYNNEOS has more limited supply but can be administered to more people.”	111 (28.9)	13 (35.1)	98 (28.2)
Alternative treatment methods	Mentions alternative mpox treatment (ie, herbal remedies, homemade remedies, and foods) either in text or reflected in the image.	“they have mentioned making sure that you stay hydrated” “make sure your body is clean and safe” “use soap and water or antibiotic ointment”	2 (0.2)	0 (0)	2 (0.2)
Traditional biomedical treatment	Mentions traditional biomedical mpox treatment (ie, antiviral treatments, such as tecovirimat [TPOXX], etc) either in the text or reflected in the image.	“The antiviral medication tecovirimat (TPOXX) is used to treat patients at risk for severe diseases who have painful lesions”	25 (2.5)	3 (3)	21 (2.3)
Benefits of treatment (n=25)	Mentions the benefits of a treatment (ie, smallpox antiviral drugs may be used to treat monkeypox)?	“While there are no specific treatments for monkeypox infections, antiviral drugs licensed for smallpox use are effective and can be used against monkeypox. These might be advised for people more likely to get very ill, including those with weak immune systems.”	8 (32)	1 (25)	7 (33.3)
Barriers to treatment (n=25)	Mentions barriers to treatment of mpox (ie, antiviral meds not accessible).	“While there are no specific treatments for monkeypox infections, antiviral drugs licensed for smallpox use are effective and can be used against monkeypox. These might be advised for people more likely to get very ill, including those with weak immune systems.”	7 (28)	0 (0)	7 (33.3)
Prevalence	Mentions the prevalence of mpox (eg, “1 in 100 people are infected” or “50 cases have been found in Indiana”).	“There are now over 31,000 cases of Monkeypox reported globally, with over 9400 cases reported in the United States.”	271 (27.1)	44 (44)	227 (25.2)
Stigma	The post is negative toward or stigmatizing of some priority population (eg, gay and bisexual men).	“Don’t have sex with your gay friend’s dog.”	43 (4.3)	6 (6)	37 (4.1)
Sources cited	The post cites a source for mpox information? (eg, CDC^e^, WHO^f^, a doctor, peer-review journal, etc. It is okay to indicate “yes,” if the post originator is citing itself, as long as it is a clear citation.)	“Listen as Dr Makary shares the reality of the situation.”	344 (34.4)	49 (49)	295 (32.8)

a Not applicable.

b GIFs: graphics interchange formats.

c IGTV: Instagram TV.

d LGBTQ+: lesbian, gay, bisexual, transgender, and queer/questioning.

e CDC: Centers for Disease Control and Prevention.

f WHO: World Health Organization.

#### Location

A total of 622 posts included a geographical tag of some kind (Table 3). Of the 622 posts, 41.8% (n=260) geotagged a location outside of the United States. Among US-based posts, the top 10 most commonly represented states were California (n=66), New York (n=60), Michigan (n=30), New Jersey (n=24), Texas (n=20), Illinois (n=19), Georgia (n=17), North Carolina (n=12), Florida (n=10), and Massachusetts (n=9).

#### Hashtag Analysis

A total of 589 posts contained at least one hashtag. The most prevalent hashtag found across posts was #monkeypox, appearing roughly 8 times more frequently than the next most prevalent hashtags (ie, #health and #monkeypoxvirus). Other health-related terms used ranged from “#virus,” “#who,” and “#health” to other conditions like “#covid19” and sexuality-related tags such as “#lgbtq.” Less than half (414/1000; 41.4%) of posts mentioned mpox by name, while the subsequent hashtags were referenced on much smaller portions of the overall sample. The top 10 most used hashtags are displayed in Table 4.

**Table 4.** Ten most common hashtags found in mpox-related Instagram posts (n=589 containing hashtags).

Hashtag	Count and prevalence, n (%)
#monkeypox	398 (67.6)
#health	48 (8.1)
#monkeypoxvirus	47 (8)
#covid19	40 (6.8)
#who	38 (6.5)
#news	37 (6.3)
#virus	34 (5.8)
#publichealth	30 (5.1)
#coronavirus	28 (4.8)
#lgbtq	26 (4.4)

### RQ2: What Mpox Causes and Solution Framing Are Conveyed in Instagram Posts (ie, What Is the Content on Instagram Related to Mpox Causes, Treatment, and Prevention)?

Regarding solution framing, just over one-third (384/1000, 38.4%) of posts promoted at least one prevention method, most commonly the mpox vaccine (263/384, 68.5%), followed by avoiding close contact (56/384, 14.5%), avoiding contact with objects (32/384, 8.3%), and abstaining from sexual activity (29/384, 7.6%) and condom use (5/384, 1.3%). A sizable number of prevention-related posts highlighted barriers to prevention methods (111/384, 28.9%).

A very small percentage of the sample mentioned mpox treatments (25/1000, 2.5% mentioned traditional biomedical treatments, such as antiviral medication; 2/1000, 0.2% mentioned alternative treatment methods). Of those posts that were coded as having depicted traditional biomedical treatments for mpox (n=25), about one-third mentioned the benefits of treating it (eg, “antiviral drugs licensed for smallpox use are effective and can be used against monkeypox”). Among these same posts, 28% (7/25) mentioned barriers to receiving treatment (eg, “WHO does not recommend vaccinating against mpox” and “there is currently a limited supply of JYNNEOS, also known as Immune or Imvanex”).

Due to poor interrater reliability regarding depictions of mpox causes on Instagram, this element of the research question was not directly addressed. However, the prevention variables do capture aspects of risk, such as close exposure to infected persons (eg, skin-to-skin and exchange of bodily fluids) and objects used by infected persons.

### RQ3: What Are the Content Characteristics of the Top 10% Most-Liked Posts on Mpox Within the Sample?

Statistically significant differences were identified between the top 10% most-liked posts and the rest of the posts in the sample. One of the most notable and expected differences is that the top 10% most-liked posts primarily originated from public figures or celebrities (MD=−0.591; *P*<.001) if they originated from an individual’s account, compared to the rest of the posts. Among organizational accounts, the top 10% most-liked posts were more likely to be coded as a business (MD=−0.299; *P*<.001) or news/media organization (MD=−0.350; *P*<.001). Among the bottom 90% liked posts, posts were more likely to originate from official governmental accounts (MD=0.222; *P*<.001), nonprofit organizations (MD=0.129; *P*=.006), and health care organizations (MD=0.102; *P*=.005).

A statistically significant higher proportion of the top 10% most-liked posts included a nonmoving photo/image in their posts, as opposed to the rest of the posts (MD=−0.119; *P*=.024), included visual depictions of mpox on body parts (MD=−0.081; *P*=.035), mentioned prevalence of mpox (MD=−0.18; *P*<.001), and cited sources (MD=−0.162; *P*=.001).

## Discussion

### Main Context

This study aimed to evaluate the content and source characteristics of mpox-related posts on Instagram. Broadly, our results highlight the quantity and diversity of health-related content that can be found online, including the range of content types, sources, and message framing within mpox-related Instagram posts. We contextualize our findings in relation to the existing academic literature below.

### Mpox Prevention and Treatment Posts Highlight the Utility of Social Media for Health Communication

Social media maintains an unfortunate reputation as a harbinger of misinformation, social anxiety, and political divide [15]. Numerous studies have highlighted that increased polarization in the United States, and the rise of misinformation can largely be attributed to excessive social media use and poor content moderation [16]. However, other studies have highlighted that, in a health communication context, social media can potentially transcend numerous cultural, structural, and systemic barriers to care to promote knowledge and improve health outcomes [17]. This observation has been previously made in computational and qualitative studies pertaining to addiction and recovery [18], pre-exposure prophylaxis for HIV prevention [19], reproductive decision-making, and vaccine communication [20]. Collectively, these studies have observed that helpful content can offset poorly vetted or low-quality information. However, certain criteria must be met, including evidence of a post originating from a verified account, culturally adaptive messaging, and the presence of online, real-time moderators to mitigate the risk of misinformation.

Our data largely contain evidence of accurate and insightful health promotion along the aforementioned criteria. For instance, within the top 10% of posts, much of our sample was coded as originating from a business entity or news organization. These posts were interpreted as part of information dissemination efforts during the early stages of the global mpox outbreak. In addition, we observed a substantive minority of posts related to treatment and prevention methods, most of which aligned with the standards set by the CDC and other similar entities. Importantly, our sample did not include hateful, vitriolic, or deliberately misinformed content about mpox. While this does not negate the potential existence of such content on Instagram, our findings suggest that, among highly engaged content, the information appears to be generally useful for individuals seeking answers about mpox risk, infection, spread, and treatment. Future research should consider expanding the scope to a larger sample of posts, which could help in identifying more generalizable findings or conducting a deeper qualitative review for information quality.

Broadly, our findings align with those of previous research illustrating the potential benefits of social media for cost-effective health communication and information sharing. For example, Jiang et al [21] highlighted that, despite known risks of social media and information quality, social media should play a continued and growing role in health communication for health promotion. As Valdez and Patterson [22] contended, the applicability of social media for health promotion will depend on the role of content moderation. On Reddit, for example, posts pertaining to health are highly moderated by trained professionals, which result in constructive and helpful messaging pertaining to myriad health behaviors, including those pertaining to sexual and reproductive health. Although it is unclear the extent to which Instagram is moderated by such professionals, our results at least illustrate that the platform, and likely the Metaverse more broadly, may be creating such avenues for informed health content. However, we acknowledge that caution must be undertaken given documented research citing Instagram’s photo-sharing capability as a venue for body image concerns.

### Comparison With Prior Work

Empirical work examining mpox on Instagram remains scarce. One study analyzing posts under #monkeypox documented early themes and misinformation risks, noting that stigmatizing and inaccurate narratives coexisted with public health messaging on the platform [22]. Compared with that work, our analysis uses a substantially larger randomized sample, a validated multidomain codebook adapted from prior Instagram health studies, and a direct comparison of the top 10% most liked posts to the remaining corpus, enabling inference about engagement-linked content characteristics (eg, source types, image features, prevalence mentions, citations).

Findings from adjacent platforms converge on several patterns that our study also observes. On TikTok, for example, multiple analyses reported variable information quality and prominent engagement with visually compelling content; videos created by physicians or authorities were relatively limited; and misinformation or conspiracy content appeared early in the outbreak, especially in trending feeds [23,24]. On X, formerly known as Twitter, studies have documented high volumes of mpox discourse with substantial misinformation and stigma toward LGBTQ+ communities, alongside network dynamics where news outlets and a small number of influencers shaped attention [11,25]. The analyses of Reddit have emphasized community problem-solving and information-seeking among gay and bisexual- and men who have sex with men–focused subreddits, often moderated to reduce misinformation and toxicity, with themes evolving as the outbreak and public health declarations unfolded [26]. Cross-platform work comparing Reddit and X found topic and psycholinguistic differences that map onto platform cultures—more community support and practical guidance on Reddit versus faster, more event-driven discourse on X [27]. Broader social media studies during the 2022 mpox emergency also traced toxicity dynamics and the need for targeted mitigation strategies, reinforcing the public health value of verified source cues and active moderation [28].

Against that backdrop, our Instagram results add 3 contributions: information regarding source composition, content features associated with engagement, and tone and quality signals. First, we show that highly engaged Instagram posts disproportionately originate from public figures/celebrities and news/business accounts, while government, nonprofit, and health care organizations populate the long tail—mirroring influence concentrations on X but documented here for Instagram with a larger coded sample. Second, nonmoving images (vs videos), visible depictions of lesions, explicit prevalence mentions, and the presence of citations all characterize the top-liked posts; prior TikTok and X studies did not simultaneously test this combination of features on Instagram. Third, unlike some cross-platform studies that reported on misinformation and stigma, our sample of highly engaged Instagram posts contained comparatively little hateful or deliberately misleading content, suggesting that—at least during the window we studied—Instagram’s most amplified mpox content skewed toward broadly informative material. This aligns with municipal and public health agency experiences that consistent, branded messaging can perform effectively on real-time platforms when paired with clear visuals and timely updates [29].

### Limitations, Strengths, and Future Research Recommendations

This study is not devoid of limitations. First, we were only able to collect information from publicly available Instagram accounts. Second, this study intentionally focused on the initial outbreak period (May 2022 to January 2023). Accordingly, an area for future research might incorporate more recent data. Third, our inclusion criteria may have limited the generalizability of our results. For example, we only included Instagram content written in English. Fourth, we acknowledge that likes are influenced by account size and reach. While engagement rates were not available via CrowdTangle, we interpret the findings as reflecting visibility rather than normalized engagement. Finally, given multiple statistical tests, findings should be interpreted cautiously as exploratory.

Despite these limitations, this study has several strengths and implications. First, this research remains the largest of its kind, to our knowledge, examining mpox on Instagram, one of the most used social media platforms in the United States. Only one other research study has examined mpox on Instagram, and that research focused on a smaller number of posts and was limited to assessing misinformation [30]. To date, a few other studies have examined mpox content via other social media platforms, including Reddit [31] and X [11,32]. Given that mpox-related posts by public figures and celebrities were among the most liked in this sample, medical experts could partner with celebrities and influencers to lead awareness campaigns on mpox.

### Conclusions

Instagram has functioned as a consequential channel in the mpox information ecosystem. In a large, randomized sample of posts spanning the initial 2022 to 2023 outbreak period, we found that organizational accounts—especially news/media and businesses—and public figures disproportionately produced the most-liked content; visual stills, lesion depictions, prevalence statistics, and explicit citations were each associated with higher engagement; and prevention and treatment content—when present—generally aligned with authoritative guidance.

Taken together, these findings imply that public health communicators can improve Instagram reach and relevance by packaging guidance as clear static visuals (eg, infographics and single-image posts) that include succinct prevalence/context cues, citing sources (eg, WHO) prominently in the caption or image, and partnering with trusted creators and news accounts to copublish updates and frequently asked questions. Given cross-platform evidence of misinformation, stigma, and toxicity—particularly on TikTok and X—strategies that foreground verified provenance, rapid myth-busting, and community-informed moderation remain essential [23].

Finally, our engagement-contrast design suggests a practical playbook for future outbreaks: monitor which content attributes travel on each platform and then rapidly iterate message formats and partnerships accordingly. Future work should extend this analysis to reels and video formats, test message experiments (A/B) with creator collaborations, and link post-level exposures to downstream behaviors (eg, vaccine information-seeking and clinic clicks). As emergent mpox developments continue to surface—and new emergencies arise—Instagram can be leveraged as a scalable, equity-minded conduit for timely, accurate risk communication when campaigns adopt these evidence-based practices.

## Supplementary material

10.2196/85379 Multimedia Appendix 1 Codebook.

## Abbreviations

CDC: Centers for Disease Control and Prevention
LGBTQ+: lesbian, gay, bisexual, transgender, and queer/questioning
MDs: mean differences
WHO: World Health Organization
