# Supplementary material for: Mpox on Instagram: Content Analytic Study
Source: JMIR Infodemiology. 2026 Jun 30;6:e85379. doi: 10.2196/85379 (PMC13318204; doi:10.2196/85379)
Supplement: Multimedia Appendix 1 [file infodemiology-v6-e85379-s001.docx]

**Appendix: Codebook**

| Element | Variable names (Cohen’s Kappa, if applicable) | Coded theme | Code | Definition | Source |  |
| --- | --- | --- | --- | --- | --- | --- |
| Inclusion | Include (N/A) | Inclusion | No = 0  Yes = 1 | Does the post meet inclusion criteria? (English language and mentions human mpox in some capacity anywhere in the post or image (this can be in an Infographic and not specified in text). |  |  |
| RQ1: What are the source and content characteristics of posts related to mpox? | | | | | Adapted from Tang & Park (2017) |  |
| Source characteristics  (Profile Type) | ProfileType (0.90) | Profile Type | Individual = 1  Organization = 2  Other (Please describe) = 3 | 1. Profile represents a single person, not an organization or a cause. 2. Profile represents an organization, not a single person. 3. Profile represents a profile other than an individual or organization | Walsh-Buhi et al (2021) |  |
| Source location | Location (0.66)  GeotaggedTXT | Location | No=0  Yes=1  If yes, fill in GeotaggedTXT with exact geotag | Location is specified anywhere in the post, either geotagged or referenced in text. | New |  |
| Source characteristics – Organization  (When referencing profile bio, you may click link to confirm organization classification)  *Check all that apply* | Business (0.63) | Business Entity | No = 0  Yes = 1 | *Display if ProfileType = 2*  Profile explicitly mentions a company, franchise, business, store, product, or service. ‘Treating patients’ qualifies as a service. The word ‘.com’ may be a qualifier. Exclude non-profit and government. | Walsh-Buhi et al (2021) |  |
|  | Nonprofit (0.74) | Advocacy/Non-Profit Group | No = 0  Yes = 1 | *Display if ProfileType = 2*  Profile represents an organization that advocates for a specific cause (‘mpox prevention’). The word ‘foundation’, mention of 501c3, ‘non-profit’, and ‘.org’ are qualifiers. Exclude business and government. | Walsh-Buhi et al (2021) |  |
|  | Government (0.87) | Government | No = 0  Yes = 1 | *Display if ProfileType = 2*  Profile represents city, state, or federal government agency. “.gov” is a qualifier. Exclude business and government. | Walsh-Buhi et al (2021) |  |
|  | News (0.91) | News/Media Organization | No = 0  Yes = 1 | *Display if ProfileType = 2*  Profile represents a news organization or type of media outlet. Media can be television, print, or digital. Mention of ‘Network’ or ‘providing news’ isn’t enough to qualify on its own. (e.g., CNN, New York Times, Wall Street Journal) | Walsh-Buhi et al (2021) |  |
|  | School (0.92) | School | No = 0  Yes = 1 | *Display if ProfileType = 2*  Profile represents a school or school district. Cannot be an online or for-profit school. The word ‘affiliate’ of X school and ‘.edu’ are qualifiers. | Walsh-Buhi et al (2021) |  |
|  | HlthCareOrg (0.71) | Healthcare Organization | No = 0  Yes = 1 | *Display if ProfileType = 2*  Profile represents a healthcare establishment that is tangible. (Hospital, clinic). This can also be coded as Business or Non-Profit in addition to CareOrg. | Walsh-Buhi et al (2021) |  |
| Source characteristics – Individual  *Check all that apply* | Parent (0.85) | Mother/Father | No = 0  Yes = 1 | *Display if ProfileType = 1*  Any mention in the post being reviewed OR on the user profile of being a mother or father. | Walsh-Buhi et al (2021) |  |
|  | Child (0.95) | Son/Daughter` | No = 0  Yes = 1 | *Display if ProfileType = 1*  Any mention in the post being reviewed OR on the user profile of being a son or daughter. | Walsh-Buhi et al (2021) |  |
|  | Journalist (0.90) | Journalist | No = 0  Yes = 1 | *Display if ProfileType = 1*  Any mention in the post being reviewed OR on user profile of a news organization, or to being a press member/journalist. | Walsh-Buhi et al (2021) |  |
|  | Physician (0.95) | Doctor | No = 0  Yes = 1 | *Display if ProfileType = 1*  Any mention in the post being reviewed OR on user profile of being a medical doctor, M.D, D.O, or medical school resident. | Walsh-Buhi et al (2021) |  |
|  | Epi (0.95) | Epidemiologist | No = 0  Yes = 1 | *Display if ProfileType = 1*  Any mention in the post being reviewed OR on user profile of being an Epidemiologist (not doctor, health educator or health worker. | Walsh-Buhi et al (2021) |  |
|  | HlthEduc (0.95) | Health educator | No = 0  Yes = 1 | *Display if ProfileType = 1*  Any mention in the post being reviewed OR in the post being reviewed OR on user profile of being a health educator (not doctor or nurse). | Walsh-Buhi et al (2021) |  |
|  | HlthWorker (0.95) | Nurse/Other Health Worker | No = 0  Yes = 1 | *Display if ProfileType = 1*  Any mention on user profile of being a nurse or health worker of any kind (not doctor or health educator). | Walsh-Buhi et al (2021) |  |
|  | PublicFigure (0.85) | Celebrity/influencer | No = 0  Yes = 1 | *Display if ProfileType = 1*  User profile is categorized as a public figure. Minimum of 20,000 followers. Any verified account would also be included. | New |  |
|  | Educator (0.95) | Teacher/educator/school official | No = 0  Yes = 1 | *Display if ProfileType = 1*  Any mention on user profile of being a teacher, educator, or other school official, but the account appears to be created in their personal capacity. | New |  |
| Content - Information about mpox | Photo (0.90) | Photo provided as media | No = 0  Yes = 1 | Post includes some sort of photo (non-moving photo image, snapshot). | Walsh-Buhi et al (2021) |  |
|  | ImageText (0.90) | Infographic provided as media | No = 0  Yes = 1 | Post includes some sort of infographic (photos, graphics, or illustrations with factual information included on them, (e.g., charts). | Walsh-Buhi et al (2021) |  |
|  | Video (0.79) | Video provided as media | No = 0  Yes = 1 | Post includes some sort of video (e.g., Boomerangs, gifs, IGTV). | Walsh-Buhi et al (2021) |  |
|  | RaceTag (1.0); RaceTXT (N/A) |  | No = 0  Yes = 1  If yes, copy and paste | Post includes race-associated tags (e.g., #Black, #Latino) or any mention of race in the text of the post. | Walsh-Buhi et al (2021) |  |
|  | GenderTag  MaleTagTXT (N/A)  FemaleTagTXT (N/A)  OtherGenderTXT (N/A) |  | No = 0  Yes= 1 | Post includes gender-associated tags (e.g., "man," "boy," "woman," "girl," "trans," "transgirl,"). If yes, copy and paste text. | Adapted from Muralidhara & Park (2018) |  |
|  | SexualityTag (1.0)  SexualityTXT (N/A) |  | No = 0  Yes= 1 | Post includes sexuality tags (e.g.,”gay,” “homosexual,” “msm” [men who have sex with men), “LGBTQ+”]). If yes, copy and paste text |  |  |
| Content- Photo characteristics | BP_Face/Head (1.0)  BP_Neck (1.0)  BP_Chest (1.0)  BP_Arms (0.66)  BP_Hands (1.0)  BP_Legs (1.0)  BP_Genitalia (1.0) | Body Part | No = 0  Yes = 1 | Image displays mpox on a specific body part, i.e. face, chest, arms, hands, legs, genitalia. | New |  |
|  | | Model (0.69) | Model | No = 0  Yes = 1 | Image displays a person or persons in photo. | New |
|  | Monkey (1.0) | Monkey photo | No = 0  Yes = 1 | Image displays a monkey or monkeys in photo. | New |  |
| RQ2: What mpox causes and solution framing are conveyed in Instagram posts (that is, what is the content on Instagram related to mpox causes, treatment, and prevention)? | | | | | Adapted from Tang & Park (2017) |  |
| Content- Risk factors (cause) | MP_Risk | Mpox Risk | No = 0  Yes = 1 | Does the post mention mpox risk (cause) in general? |  |  |
|  | MP_Risk_1 (1.0)  MP_Risk_2 (1.0)  MP_Risk_3 (0.0)  MP_Risk_4 (0.0)  MP_Risk_5 (0.0)  MP_Risk_6 (0.24)  MP_Risk_7 (-0.18)  MP_Risk_7_TXT MP_Risk_9 (N/A)  MP_Risk_10 (N/A) | Mpox Risks:  1_Respiratory secretions  2_Skin lesions  3_Contaminated objects  4_Droplet respiratory particles  5_During birth [congenital mpox]  6_Sexual transmission  7_Other  9_Close contact with someone who tested positive  10_ General exposure | No = 0  Yes = 1 | *Display if Risk=1*  Does post mention mpox risk factors? (There should be an explicit mention of risk due to close contact with respiratory secretions, skin lesions, recently contaminated objects, droplet respiratory particles, during birth [congenital mpox], and likely through sexual transmission) | [https://www.who.int/news-room/fact-sheets/detail/mpox](https://www.who.int/news-room/fact-sheets/detail/monkeypox) |  |
| Content- Prevention  Primary | Prevention (0.80) | Prevention | No = 0  Yes = 1 | Does the post mention mpox prevention methods in general? |  |  |
|  | Condoms (0.80) | Promotes condom use | No = 0  Yes = 1 | *Display if Prevention=1*  Post promotes the use of condoms as a form of mpox prevention. | https://www.cdc.gov/poxvirus/mpox/prevention/sexual-health.html |  |
|  | Vaccination (0.72) | Promotes vaccination | No = 0  Yes = 1 | *Display if Prevention=1*  Post promotes getting the vaccine as a form of mpox prevention. | https://www.cdc.gov/poxvirus/mpox/prevention/protect-yourself.html |  |
|  | Contact (0.72) | Avoid close contact with infected persons | No = 0  Yes = 1 | *Display if Prevention=1*  Post promotes avoiding close contact with infected persons as a form of mpox prevention. | https://www.cdc.gov/poxvirus/mpox/prevention/protect-yourself.html |  |
|  | Abstinence (0.72) | Promotes avoiding sexual contact | No = 0  Yes = 1 | *Display if Prevention=1*  Post promotes abstaining from kissing, hugging, cuddling, and any other sexual activity as a form of mpox prevention. | https://www.cdc.gov/poxvirus/mpox/prevention/sexual-health.html |  |
|  | Objects (0.81) | Promotes avoiding contact with objects | No = 0  Yes = 1 | *Display if Prevention=1*  Avoiding contact with objects (including bedding, sex toys, towels, clothing) or sharing food/drink/other that an infected person has touched/used. | https://www.cdc.gov/poxvirus/mpox/prevention/protect-yourself.html |  |
| Treatment | Treatment (0.0) | Mentions some forms of therapeutic or treatment | No = 0  Yes = 1 | Post mentions some form of therapeutic/clinical care or treatment for people infected with mpox. | https://www.who.int/news-room/fact-sheets/detail/mpox |  |
|  | AltTreat (1.0) | Promotes alternative treatment | No = 0  Yes = 1 | Mentions alternative mpox treatment (i.e., herbal remedies, homemade remedies, foods) either in text or reflected in the image. | Need a source  https://www.earthclinic.com/cures/mpox.html |  |
|  | TradTreat (1.0) | Promotes traditional biomedical treatment | No = 0  Yes = 1 | Mentions traditional biomedical mpox treatment (i.e., Antiviral treatments, such as TPOXX, etc.) either in the text or reflected in the image. | https://www.who.int/news-room/fact-sheets/detail/mpox |  |
| Content – Diagnosis, treatment, and prevention | Diagnosis (0.0) | Diagnosis | No = 0  Yes = 1 | Mentions mpox diagnostic methods (i.e., Biopsy, PCR test from lesion swab sample) | https://www.who.int/news-room/fact-sheets/detail/mpox |  |
| Content- Prevalence | Prevalence (0.90) | Mentions the prevalence/ susceptibility of mpox | No = 0  Yes = 1 | Post mentions the prevalence of mpox i.e., “1 in 100 people are infected” or “50 cases have been found in Indiana”). | Tang & Park (2017) |  |
| Content- Severity | Seriousness (0.0) | Mentions how serious mpox could be | No = 0  Yes = 1 | Post mentions the seriousness of mpox in terms of medical consequences (death), financial consequences (high cost of treatment), or other types of consequences. | Tang & Park (2017) |  |
| Content- Benefits | PrevBenefit (0.72) | Mentions the benefit of a prevention method | No = 0  Yes = 1 | *Display if Prevention=1*  The post mentions the benefits of a prevention method (e.g., condoms also protect against sexually transmitted infections, pregnancy, etc.). | Adapted from Tang & Park (2017) |  |
|  | DiagBenefit (0.0) | Mentions the benefit of a diagnostic method | No = 0  Yes = 1 | *Display if Diagnosis=1*  The post mentions the benefits of diagnostic methods (i.e., Swab/specimen test). | Adapted from Tang & Park (2017) |  |
|  | TreatBenefit (1.0) | Mentions the benefit of a treatment | No = 0  Yes = 1 | *Display if Treatment=1*  The post mentions the benefits of a treatment (i.e., Smallpox antiviral drugs may be used to treat mpox). | Adapted from Tang & Park (2017) |  |
| Content - Barriers | DiagBarrier (0.0) | Mentions the barriers of diagnostic methods | No = 0  Yes = 1 | *Display if Diagnosis=1*  The post mentions barriers to diagnostic mpox methods (i.e., lack of knowledge on what mpox looks like, lack of skilled or trained physicians, lack of testing). | Adapted from Tang & Park (2017) |  |
|  | TreatBarrier (1.0) | Mentions barriers of treatment | No = 0  Yes = 1 | *Display if Treatment=1*  The post mentions barriers to treatment of mpox (i.e., Antiviral meds not accessible). | Adapted from Tang & Park (2017) |  |
|  | PrevBarrier (0.71) | Mentions barriers of prevention | No = 0  Yes = 1 | *Display if Prevention=1*  The post mentions barriers to the prevention of mpox (i.e., lack of knowledge on prevention methods, vaccines are unavailable). | Adapted from Tang & Park (2017) |  |
| Content- HBM | CueAction (0.27) | Includes a cue to action | No = 0  Yes = 1 | The post includes information that urges readers to adopt a certain behavior (eg. “abstain from sex with exposed individuals”, “Keep your distance from someone who you think has mpox”). | Adapted from Tang & Park (2017) |  |
|  | Stigma (1.0) | Stigma | No = 0  Yes = 1 | The post is negative towards or stigmatizing of some priority population (e.g., gay and bisexual men). |  |  |
| Content - Source attribution | Cite (0.47) | Citation included | No = 0  Yes = 1 | The post cites a source for mpox information? (e.g., CDC, WHO, a doctor, peer-review journal, etc. It is okay to indicate "yes," if the post originator is citing itself, as long as it is a clear citation.) | Walsh-Buhi et al (2021) |  |
|  | GovInfo (0.87) | Cites the CDC | No = 0  Yes = 1 | *Display if Cite=1*  The post cites information from the CDC or other federal level or foreign equivalent sources. | Walsh-Buhi et al (2021) |  |
|  | DoctorInfo (0.47) | Cites a doctor | No = 0  Yes = 1 | *Display if Cite=1*  The post cites information from a medical doctor (e.g., Dr. Martinez, a local pediatrician). Announcing a talk by a doctor is not sufficient. | Walsh-Buhi et al (2021) |  |
|  | PoliticalInfo (0.47) | Cites political officials or political organizations | No = 0  Yes = 1 | *Display if Cite=1*  The post cites information from government officials (e.g., senators, governors, representatives) or from political organizations (e.g., Focus on the Family). | Walsh-Buhi et al (2021) |  |
|  | ResearchInfo (0.47) | Cites research community | No = 0  Yes = 1 | *Display if Cite=1*  The post cites information from a member of the research community (e.g., some university, researchers, scientists, epidemiologists, professors). | Walsh-Buhi et al (2021) |  |
|  | OtherWebInfo (0.47) | Cites WebMD or other health/web source | No = 0  Yes = 1 | *Display if Cite=1*  Other post source such as WebMD, Mayo Clinic, etc. (not CDC). | Walsh-Buhi et al (2021) |  |
|  | HealthDeptInfo (0.47) | Cites state or local health department | No = 0  Yes = 1 | *Display if Cite=1*  The post cites information from a state or local health department. | Walsh-Buhi et al (2021) |  |
|  | WHOInfo (0.47) | Cites WHO | No = 0  Yes = 1 | *Display if Cite=1*  The post cites information from the World Health Organization (WHO) | Walsh-Buhi et al (2021) |  |
| Content - Personal account | Personal (1.0) | Personal account | No = 0  Yes = 1 | Post has presence of personal account from individuals who mentioned a firsthand experience with mpox (Only from individual posters; e.g., poster said *they* have or had mpox). | Walsh-Buhi et al (2021) |  |
|  | Secondhand (1.0) | Secondhand account | No = 0  Yes = 1 | Post has presence of personal account from individuals who mentioned a secondhand experience with mpox (Only from individual posters; e.g., poster said they are/were a close contact or know someone who has/had mpox). |  |  |
|  | Hashtag (N/A) |  |  | Copy and paste all hashtags from the post. (Check also for account owner comments that include additional hashtags; do not include comments by others.) | Walsh-Buhi et al (2021) |  |
| RQ5: What are characteristics of the contents of the top 10% most liked posts on mpox within the sample? | | | | | Adapted from Tang & Park (2017) |  |

**The variables in the codebook were guided by the following references:**

1. Tang L, Park S-E. Sun Exposure, Tanning Beds, and Herbs That Cure: An Examination of Skin Cancer on Pinterest. Health Commun 2017 Oct;32(10):1192–1200. [doi: 10.1080/10410236.2016.1214223]

2. Walsh-Buhi E, Houghton RF, Lange C, Hockensmith R, Ferrand J, Martinez L. Pre-exposure Prophylaxis (PrEP) Information on Instagram: Content Analysis. JMIR Public Health Surveill 2021 Jul 27;7(7):e23876. PMID:34061759

3. Basch CH, Hillyer GC. Skin cancer on Instagram: implications for adolescents and young adults. Int J Adolesc Med Health 2020 Feb 7;/j/ijamh.ahead-of-print/ijamh-2019-0218/ijamh-2019-0218.xml. PMID:32031976

4. Muralidhara S, Paul MJ. #Healthy Selfies: Exploration of Health Topics on Instagram. JMIR Public Health Surveill 2018;4(2):e10150. [doi: 10.2196/10150]

5. Park S-E, Tang L, Bie B, Zhi D. All pins are not created equal: communicating skin cancer visually on Pinterest. Transl Behav Med 2018 Apr 17;9. [doi: 10.1093/tbm/iby044]

6. Jhawar N, Lipoff JB. Variable potential for social media platforms in raising skin cancer awareness. Dermatol Online J 2019 Jun 15;25(6):13030/qt2t78m4x1. PMID:31329387
